# Editorial: Education and training in pharmaceutical medicine, medicines development and regulation for scientists worldwide

**DOI:** 10.3389/fphar.2025.1544678

**Published:** 2025-03-18

**Authors:** Bernd Rosenkranz, Tomoya Tachi, Rolf Bass

**Affiliations:** ^1^ Fundisa African Academy of Medicines Development, Cape Town, South Africa; ^2^ Institute for Clinical Pharmacology and Toxicology, Charité Universitätsmedizin Berlin, Berlin, Germany; ^3^ Division of Clinical Pharmacology, Faculty of Medicine and Health Sciences, Stellenbosch University, Stellenbosch, South Africa; ^4^ Department of Clinical Pharmacy, Graduate School of Pharmaceutical Sciences, Nagoya City University, Nagoya, Japan

**Keywords:** education, training, pharmaceutical industry, graduate programs, professional development courses, pharmaceutical medicine, medicines development, medicines regulation

The effectiveness of medical treatments and other interventions are assessed to optimize outcomes and healthcare delivery to enable clinicians, patients, healthcare providers and managers, funders, policymakers and government to make informed decisions (Rosenkranz, 2024). The development and regulatory review of novel (better) medicines that are safe, effective and of good quality have thus become increasingly complex over the past decades, with the establishment of new technologies and concepts and the interdisciplinary and global nature of the pharmaceutical industry.

The need for effective capacity strengthening in medicines development and regulation has been recognized worldwide, both in first-world as well as developing and low- and middle-income countries (ESSENCE on Health Research and CCR, 2023). Educational and training programs have been developed by Universities or other organizations (for example: Semete-Makokotlela et al., 2021; Najjemba et al., 2023; Kerpel-Fronius et al., 2015). This Research Topic provides an overview of some of these initiatives, their opportunities and challenges.

Mollet et al. outline the development and current activities of the European Center of Pharmaceutical Medicine (ECPM) which has been established as non-for-profit institution in 1991, together with the University of Basel Department of Public Health. This program has been recognized as a PharmaTrain Center of Excellence for 12 consecutive years and is now also offered in the United States, China and India. Relationships with key players in the field assist in defining the course content and in securing expert speakers. Digital technologies have allowed for great flexibility.

Another project which is aligned with the Innovative Medicines Initiative (IMI) PharmaTrain project (16 IMI Call 2008/1/16) is the pharmaceutical medicine course at Semmelweis University Budapest, Hungary (Kerpel-Fronius et al.). Its aim is to extend training in medicines development to those EU member states where no such education existed. The program benefits from cooperation with other universities in Central and Eastern Europe in form of the Cooperative European Medicines Development Course (CEMDC). After the interruption during the COVID-19 pandemic, the program has been reorganized as a postgraduate MSc course which will start in 2025. Main educational themes are the transition from basic pharmacological to industrial research, biopharmaceutical formulation and manufacturing, and marketing aspects of medicines development.

The 7-year collaboration between King’s College London and the Global Medicines Development Professionals (GMDP) Academy is discussed by Silva et al. This blended e-learning program consists of a comprehensive curriculum based on the PharmaTrain syllabus. Course evaluation has demonstrated significant improvement in the professional activities and career progression of the participants. Another beneficial outcome of this course is the awareness of students of their identities and purposes as pharmaceutical professionals. Challenges include the digitalization, the rapid growth of artificial intelligence (US Department of Health and Human Services, 2023; WHO, 2023) and the consequences of such technologies for the curriculum and educational tools, demanding a rapid adaptation in the approaches to lifelong learning.

The development and regulatory approval of medicines for pediatric use requires special attention (Lehmann). For many medicinal products, only a limited amount of data exist on their safety and efficacy in children. This leads to the dilemma of finding a balance between protecting children and adolescents and obtaining reliable, robust and justified data to treat them adequately and not in an off-label manner with unknown risks. Education and training of all involved stakeholders is important, including healthcare professionals, industry, regulators, and legislators, but also patients and caretakers. Regulatory requirements should be harmonized.

A specific example of a significant global health condition is urothelial carcinoma (Romero-Clarà). Healthcare professionals who deal with bladder cancer patients need comprehensive training to incorporate the advances in healthcare into their clinical practice. The manuscript describes a dedicated educational program to educate healthcare professionals on bladder cancer management and on recent immunotherapy treatment options. This initiative resulted in substantial knowledge enhancement. Complementary initiatives brought together patients and medical experts to foster a holistic, patient-centered approach. The program has been successfully endorsed by regulatory bodies and professional associations.

Novel methodologies such as such as flipped classrooms and problem-based learning are used to improve student engagement and success. The introduction of a blended learning approach can lead to lower failure rates, increased student engagement, and better understanding of complex clinical pharmacology topics as demonstrated in a nursing pharmacology course in Israel (Arien-Zakay).

Training of healthcare professionals by pharmaceutical companies can result in the dissemination of biased information. An online survey amongst pharmacy students in Pakistan revealed that a significant number of them observed that promotional activities have an impact on dispensing practices and for example can contribute to irrational antibiotic prescribing (Gillani et al.). This project underscores the need for industry-independent professional education of healthcare students and professionals.

Finally, there has been a suggestion to introduce a training program on basic aspects of medicines development and regulation for Chief Executive Officers and senior management of pharmaceutical companies and related organizations to assist them in their decision making (Kramer et al.).

In conclusion, the publications assembled in this Research Topic clearly demonstrate the need for structured capacity building in medicines development and regulation using a well-designed syllabus, adequate methodologies, assessments and quality control.
